# Electronic Records, Registries, and the Development of “Big Data”: Crowd-Sourcing Quality toward Knowledge

**DOI:** 10.3389/fonc.2016.00268

**Published:** 2017-01-30

**Authors:** Summer B. Dewdney, Jason Lachance

**Affiliations:** ^1^Division of Gynecologic Oncology, Rush University Medical Center, Chicago, IL, USA; ^2^Division of Gynecologic Oncology, Maine Medical Center, Portland, ME, USA

**Keywords:** big data, cancer registry, crowd-sourcing, registries, quality measures

## Abstract

Despite many perceived advances in treatment over the past few decades, cancer continues to present a significant health burden, particularly to the aging US population. Forces including shrinking funding mechanisms, cost and quality concerns, as well as disappointing clinical outcomes have driven a surge of recent efforts into utilizing the technological innovation that has permeated other industries by leveraging large and complex data sets, so called “big data.” In this review, we will review some of the history of oncology data collection, including the earliest data registries, as well as explore the future directions of this new brand of research while highlighting some of the more recent and promising efforts to harness the power of the electronic health record and the multitude of data co-located there, in an effort to improve individualized cancer-related outcomes in rapid real time.

Both the Institute of Medicine and the American Society of Clinical Oncology (ASCO) have called for a “national quality reporting program for cancer care as part of a learning health care system” (1). Furthermore, the current Presidential administration has made cancer one of its priorities, announcing its intentions to allocate additional funds for a “moonshot” to a cure. Cancer is a major public health issue, as it is the second leading cause of death in the US and is projected to surpass/exceed heart disease in the upcoming years. The lifetime risk of developing some form of cancer for men is 42% (1 in 2) and for women 38% (1 in 3). By 2030, the incidence of cancer will rise to 2.3 million cases per year as a result of the aging US population (2).

Recent cancer research budgets have been declining; however, the current climate is much more favorable. We find ourselves at crossroads of information technology, increased funding, and increased pressure for both quality care and cures.

In pursuit of these goals, and with the atmosphere of information technology, “big data” is an unchartered area in cancer. “Big data” is the term used for data sets that are so large or complex that traditional data sets processing applications are inadequate. The formation and management of these datasets can be exploited for real-time answers both in the efficacy of treatments in the real world as well as quality of care. As payers for health care in the United States and worldwide grapple with the movement away from fee for service-based reimbursement and toward payments for quality, information gleaned from large dataset may provide feedback that is crucial for improvements in the system. Additionally, “big data” compiled for research purposes provides a real-world laboratory for innovative treatments and interventions that may, in some places, fill in gaps where randomized prospective trials are impractical or cost prohibitive.

Population-based, cancer incidence data in the United States have been collected by the National Cancer Institute’s (NCI’s) Surveillance, Epidemiology, and End Results (SEER) Program since 1973 and by the Centers for Disease Control and Prevention’s National Program of Cancer Registries (NPCR) since 1995 (2). The North American Association of Central Cancer Registries compiles and reports incidence data from 1995 onward for cancer registries that participate in the SEER program and/or the NPCR. These data approach 100% coverage of the US population in the most recent time period and were the source for the projected new cancer cases in 2016 (2). These databases have provided an invaluable resource in tracking, categorizing, and noting trends of cancer as a public health issue. However, these existing systems fail to track the quality of the care for cancer patients.

We are currently in the midst of an explosion of the information industry. However, the information technology revolution has yet to mature in the medical field, despite near-universal penetrance of the electronic medical record. Many cancer patients experience highly fragmented care, with a combination of their records on paper, different electronic health record (EHR) systems, and physical disks for imaging, each housed in multiple locations. These uncoordinated and unconnected pieces of information impair the ability of oncologists to make an impact on the population scan and more difficult for the individual patient. Research based on an EHR is limited by the complexity of data collected and the context under which the data were collected. However, the EHR has unlocked the potential to turn individual level data into datasets that can provide information about the population and the efficacy of our interventions.

To repurpose the individual electronic pieces of a patient’s electronic chart into “big data,” data models must be created using clinical, administrative, and claims data. One such dataset is the HMO Research Network Virtual Data Warehouse (VDW)—a public, non-proprietary, research-focused data model that currently consists of 17 sites that together cover 13 million individuals; in total, the VDW has over 185 million person-years of data (3). Using this VDW, Kaiser Permanente has developed clinical research networks that include a colorectal cancer cohort, a severe congenital heart disease cohort and an obesity cohort (4). It is important to establish that “big data” is different than conventional large databases, one is a system that purely collects data, whereas “big data” is harvesting the data and analyzing in a fashion that gives us real-time feedback that could help providers make decisions in patient care. This could be a turning point in our care of oncology patients if this were to be successful. The goal of “big data” is the capability to extract value from large amounts of data, not just collect it.

Over the past two decades, additional organizations have attempted to fill that quality void and establish guidelines for evidence-based cancer care. For example, the National Comprehensive Cancer Network was started in 1995 to establish practice guidelines for clinicians taking care of cancer patients. This has become an invaluable resource for clinicians. In addition, the American College of Surgeons and the American Cancer Society jointly sponsor the National Cancer Database (NCDB), which is a database that covers approximately 1,500 facilities and approximately 70% of new cancer diagnosis in the US. They have over 30 million records to date (5). In addition to the NCDB, the Commission on Cancer, which is part of the American College of Surgeons, started to use the NCDB data to establish whether institutions were meeting certain quality measures. This started with a few disease sites and now covers nine, with continued plans to broaden.

Once established, registries such as those as listed above provide insight to the epidemiology of cancer, but now with improved informational technology, we have the potential to harvest more complex and important data points. We are starting to establish quality measures and analyze them compared to recognized national benchmarks that were not available or present before. Rapid advances in health information technology have created unprecedented opportunities to learn from real-world data.

Many cancer organizations are making this a priority, including ASCO, which has included “Big Data” as one of three major visions for cancer care (6). ASCO’s CancerLinQ initiative, aims to collate data from every cancer patient in the US and make it available for analysis in the hope that it will lead to new insights. Their goals not only want to impact on a population basis but for the individual patient and provider. They propose real-time feedback to the oncologist to help them choose certain therapies and make clinical decisions. They are using a global software company, to create a big data platform. Many such software vendors are now commercially available. In the private sector, Flatiron Health has created the OncologyCloud–a big data program that aims to collect data from the medical records, doctors’ notes, and billing information, to give real-time feedback to providers about treatments and outcomes. For example, part of their analytics can analyze cost of individual patient care, identify potential clinical trial candidates, which all streamlines with their specific EMR. Another example of “big data” harvesting is the Genomic Data Commons, this was developed and is housed at the University of Chicago, and here, they are using a “big data” approach to analyze cancer genomics. They are creating a cancer research community through a unified data repository promoting precision medicine, which is sponsored by the NCI.

We are in a transition time, as technology continues to exponentially improve, soon we will be able to extract all the data and quality measures that we need directly from the EHR. The goal of big data would be to not only link current registry databases but gather all data on all cancer patients and then use to analyze outcomes, which has never been done before. But ultimately the goal of “big data” is bigger and aspirational, it not only improved quality of care but also actual answers to cancer, and improved outcomes. For example, many of the chemotherapy regimens we use today have been adopted because they demonstrated a benefit of survival in a clinical trial. Over decades, pharmaceutical trials and cooperative groups have labored through the model of expensive, lengthy trials to get answers on which chemotherapy to use in which setting. This has been the standard of how we prove drug A is better than drug B. However, this paradigm represents only a small fraction of the total number of patients with cancer (<5%). The advent of precision medicine has subdivided even common malignancies into increasing small subtypes making large prospective trials increasingly burdensome. “Big data” offers the potential to harness all of the data from all of our cancer patients. We could collect, harness, and analyze patients’ clinical information and link it to molecular data and treatment outcomes to find answers to many of cancer’s most elusive questions in real time.

The importance of health information technology in our pursuit of quality and cure cannot be underestimated. New, innovative, and affordable approaches to quality assessment and improvement as well as treatment efficacy will depend on our ability to create and maintain “big data.”

## Author Contributions

Both SD and JL wrote and edited this Perspective.

## Conflict of Interest Statement

The authors declare that the research was conducted in the absence of any commercial or financial relationships that could be construed as a potential conflict of interest.
